# Improvement of osteoporosis Care Organized by Nurses: ICON study - Protocol of a quasi-experimental study to assess the (cost)-effectiveness of combining a decision aid with motivational interviewing for improving medication persistence in patients with a recent fracture being treated at the fracture liaison service

**DOI:** 10.1186/s12891-021-04743-2

**Published:** 2021-10-29

**Authors:** Dennis Cornelissen, Annelies Boonen, Silvia Evers, Joop P. van den Bergh, Sandrine Bours, Caroline E. Wyers, Sander van Kuijk, Marsha van Oostwaard, Trudy van der Weijden, Mickaël Hiligsmann

**Affiliations:** 1 Department of Health Services Research, Care and Public Health Research Institute (CAPHRI), Maastricht University, P.O. Box 616, 6200 MD Maastricht, The Netherlands; 2 Department of Internal Medicine, Division of Rheumatology, Maastricht University Medical Center; and Care and Public Health Research Institute (CAPHRI), Maastricht, The Netherlands; 3 Centre for Economic Evaluation and Machine Learning, Trimbos Institute, Netherlands Institute of Mental Health and Addiction, Utrecht, The Netherlands; 4 Department of Internal Medicine, VieCuri Medical Center, Venlo, The Netherlands; 5 Department of Internal Medicine, Nutrition and Translational Research in Metabolism (NUTRIM), Maastricht University Medical Center, Maastricht, The Netherlands; 6 Faculty of Medicine and Life Sciences, Hasselt University, Hasselt, Belgium; 7 Department of Clinical Epidemiology and Medical Technology Assessment (KEMTA), Maastricht University Medical Center, Maastricht, The Netherlands; 8 Department of Family Medicine, Care and Public Health Research Institute (CAPHRI), Maastricht University Medical Centre, Maastricht, The Netherlands

**Keywords:** Cost-effectiveness, Process evaluation, Decision aid, Osteoporosis, Patient participation, Protocol, Medication adherence, Medication persistence, Fracture liaison service, Nurse

## Abstract

**Background:**

Given the health and economic burden of fractures related to osteoporosis, suboptimal adherence to medication and the increasing importance of shared-decision making, the Improvement of osteoporosis Care Organized by Nurses (ICON) study was designed to evaluate the effectiveness, cost-effectiveness and feasibility of a multi-component adherence intervention (MCAI) for patients with an indication for treatment with anti–osteoporosis medication, following assessment at the Fracture Liaison Service after a recent fracture. The MCAI involves two consultations at the FLS. During the first consultation, a decision aid is will be used to involve patients in the decision of whether to start anti-osteoporosis medication. During the follow-up visit, the nurse inquires about, and stimulates, medication adherence using motivational interviewing techniques.

**Methods:**

A quasi-experimental trial to evaluate the (cost-) effectiveness and feasibility of an MCAI, consisting of a decision aid (DA) at the first visit, combined with nurse-led adherence support using motivational interviewing during the follow-up visit, in comparison with care as usual, in improving adherence to oral anti-osteoporosis medication for patients with a recent fracture two Dutch FLS. Medication persistence, defined as the proportion of patients who are persistent at one year assuming a refill gap < 30 days, is the primary outcome. Medication adherence, decision quality, subsequent fractures and mortality are the secondary outcomes. A lifetime cost-effectiveness analysis using a model-based economic evaluation and a process evaluation will also be conducted. A sample size of 248 patients is required to show an improvement in the primary outcome with 20%. Study follow-up is at 12 months, with measurements at baseline, after four months, and at 12 months.

**Discussion:**

We expect that the ICON-study will show that the MCAI is a (cost-)effective intervention for improving persistence with anti-osteoporosis medication and that it is feasible for implementation at the FLS.

**Trial registration:**

This trial has been registered in the Netherlands Trial Registry, part of the Dutch Cochrane Centre (Trial NL7236 (NTR7435)).

Version 1.0; 26-11-2020.

## Background

Osteoporosis is a disease characterized by low bone mass and loss of bone quality, leading to an increased risk of fractures. Bone fractures are associated with pain, decreased mobility, reduced health- related quality of life and increased costs. Furthermore, patients with a fracture have an increased risk of subsequent fractures and premature mortality [1–6].

Fracture Liaison Services (FLS) have been encouraged to optimize post-fracture care and osteoporosis treatment [7, 8], ideally nurse led. Accumulating evidence showed that treatment at FLS leads to reduction in fractures and in potential risk of mortality, as well as improvement in medication adherence [7, 9]. However, despite improved adherence in the context of the FLS [10, 11], suboptimal levels of adherence are still common, resulting in reduced effectiveness of the anti-osteoporosis treatment, thus leading to higher rates of fracture, more subsequent fractures, lower quality of life, higher mortality and increased costs [12, 13]. Unaddressed patient values, preferences and capabilities may contribute to suboptimal adherence [7, 9, 14–18].

In recent years, several interventions have been investigated to assess their effects on adherence to anti-osteoporosis medications, e.g. education/monitoring programs, tailored interventions with counseling sessions and automatic prescriptions [19–21]. None of the interventions stood out for unequivocal effectiveness. There are indications that multi-component interventions with involvement of both patients and professionals result in better results than interventions with a single component [22–25]. The use of a decision aid (DA), i.e. involving patients in decision-making, has been put forward as an essential aspect of quality of care [21, 25–28]. However, available evidence suggests that use of a DA alone, while improving the uptake of anti-osteoporosis medication, does not improve adherence or persistence [19, 21, 29], which suggests that additional follow-up of medication-taking behavior is required. In line with this, motivational interviewing [30, 31] in osteoporosis follow-up care has promising effects on medication adherence [32–34].

To our knowledge, while promising, a combination of a DA and motivational interviewing has not yet been investigated in a FLS. Therefore, the Improvement of Osteoporosis care Organized by Nurses (ICON) study aims to evaluate the effectiveness of a multi-component adherence intervention (MCAI), consisting of nurse-led shared decision-making when anti-osteoporosis medication is indicated, combined with motivational interviewing at the follow-up visit, focusing on medication-taking behavior.

## Methods

The ICON study entails an evaluation of the intervention’s effectiveness, an economic evaluation and a process evaluation, aiming to address the following research questions:

Effect evaluation

- Is the MCAI superior in comparison with usual care in terms of persistence with oral bisphosphonates?
- Is the MCAI superior over usual care in terms of patients’ decision quality, other forms of adherence and outcomes related to patient health (e.g. fractures, mortality and quality of life)?

Economic evaluation

- From a societal perspective, what is the (lifetime) cost-effectiveness of MCAI in comparison with usual care?

Process evaluation

- Can the MCAI be performed as intended?
- What are the experiences and opinions of patients and professionals regarding MCAI and the feasibility of implementing it in FLS care?

### Design

The ICON study will be conducted within the context of the FLS. Nurses will be trained in principles of shared decision making and motivational interviewing based on a which DA has been developed for patients with osteoporosis who are starting anti-osteoporosis medication [31]. This DA will be further improved and updated according to the IPDAS guidelines [35], to be applicable for patients in the Netherlands with osteoporosis and a recent fracture, and will be adapted to current national guidelines.

We have chosen for the quasi-experimental pre-post design in which the intervention group will be included once the control group has been included completely and has completed the follow-up [36, 37]. This is the result of the organization of the FLS of which most are run by one or two osteoporosis nurse. Therefore, possible contamination in the approach by initiating the intervention prior or simultaneously with the intervention us of conducting an RCT. Once our nurses will be trained, it will be impossible to determine to what extent the training is being employed and no control patients could thus be included. This study has been approved by the Medical Ethics Committee of Maastricht University Medical Centre (MUMC+)/Maastricht University (UM), the Netherlands, registration number 2018-0575.

### Study population

Patients are eligible for study inclusion when they are 50 years old or more, visit the FLS due to a recent fracture (≤ 26 weeks), have osteoporosis (defined as a T-score ≤ − 2.5 as measured by Dual Energy X-ray absorptiometry (DXA)) and/or a moderate/severe vertebral fracture, and have not used anti-osteoporosis medication within 12 months before inclusion. Patients will be excluded if they have contra-indications for oral anti-osteoporosis medication, have severe comorbidities (e.g current malignancies), if they do not fully understand the study or are not able to fill in the questionnaires in the Dutch language.

### Setting and recruitment

The study is conducted in the FLSs of two hospitals in the southern region of the Netherlands: MUMC+, Maastricht and VieCuri Medical center, Venlo. These hospitals have been chosen because of the high volume of patients in their FLSs, their experience with conducting research, and the presence of formally qualified and dedicated osteoporosis nurses. During the first visit to the FLS, the nurse (and/or physician) informs patients briefly about the study, presenting it as a non-interventional study that aims to gain insight into the quality of post-fracture care. After this visit, patients who meet the inclusion criteria and show interest in participating are referred to the researcher or doctor’s assistant. A researcher informs the patient both verbally and in writing about the background of the study, the research burden, and safety and privacy issues. If the patient is willing to participate (patients are allowed a period of one week for consideration), the informed consent form is signed.

**Fig. 1:**
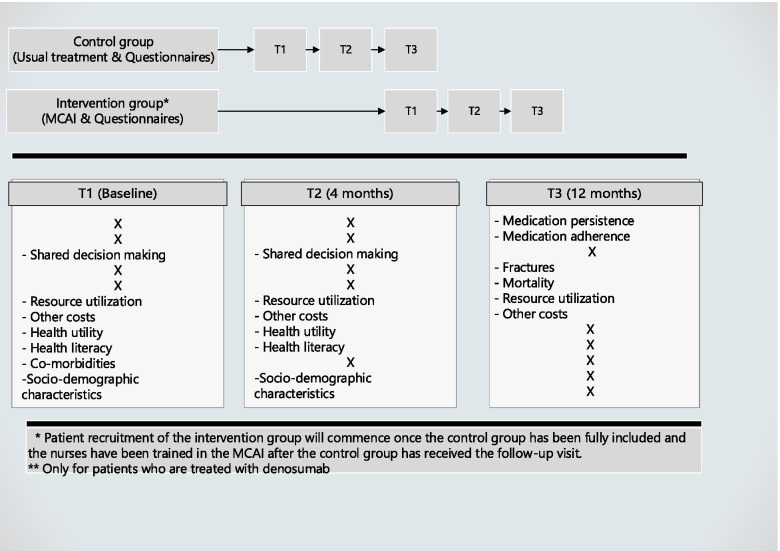
Schematic overview of the ICON study

To prevent behavioral change of patients in the control group, they do not receive any information regarding the MCAI. Patients are informed that the study aims to evaluate their post-fracture osteoporosis care. The expected time investment of the patients over the 12 months period is estimated at three hours: 30 min for the inclusion conversation and a maximum of 45 min for each questionnaire. Patients are given the option to fill out the questionnaire in a questionnaire booklet or online in the program Castor. Once the sample size for the control group is reached and all patients have received the first follow-up visit (3 to 4 months after the first visit), nurses will receive a two-day workshop on shared decision-making (in general and specifically for the DA for this study) and on motivational interviewing. Once the nurses have been trained, inclusion of patients in the intervention group will begin. The total study inclusion period of approximately 16 months is planned to accommodate the study follow-up, set at 12 months. For a schematic overview of the study, see Fig. 1.

### Sample size

Sample size for anti-osteoporosis medication persistence (i.e. the primary outcome) is based on two participating centers and calculated in the software R using the formula from Chow SC et al. [38]. Previous research has shown that approximately 43% of patients are persistent at one year when receiving usual care [39]. We consider an absolute improvement of 20% in the intervention group compared to the control group clinically relevant [40]. To obtain a power of 80% and an alpha of 5% to detect this difference, we need to include 105 patients per group when assuming 43% persistence in usual care, accounting for a potential dropout of 10%.

Although oral bisphosphonates are first line treatment according to the Dutch osteoporosis guideline, approximately 15% of the patients attending the FLS are eventually treated with other anti-osteoporotic medications, such as subcutaneous injections (i.e. denosumab and teriparatide) and intravenous infusions (i.e. zoledronic acid). To avoid treatment bias, our sample should contain only patients treated solely with oral bisphosphonates, and therefore our total sample size is increased to a total of 248 patients (124 per group).

### The multicomponent adherence intervention (MCAI)

Usual care at the FLS consists of assessment of risk for osteoporosis and for subsequent fractures, DXA and vertebral fracture assessment and laboratory assessment. In addition, further evaluation of underlying disorders and medication known to be associated with osteoporosis or fracture risk will take place. The FLS nurse summarizes the outcomes of the assessment, educates patients on the diagnosis/refracture risk, and advises on interventions with regard to lifestyle as well as anti-osteoporosis medication to improve bone health. In case of osteoporosis or presence of (asymptomatic) vertebral fractures, there is an indication for anti-osteoporosis medication, which is prescribed by the rheumatologist/endocrinologist, and further information about the intake of medication and possible side-effects will be provided by the nurse. A follow-up visit is scheduled 3 to 4 months after the initial FLS visit. The follow-up visit includes evaluation of compliance, adherence and possible side effects.

The MCAI is offered in addition to the usual care at the FLS with the following adjustments: during the first visit to the FLS, patients receive guidance in the decision on whether or not to start medication, by using a DA with the assistance of a FLS nurse. During the follow-up visit, the specialized osteoporosis nurse will inquire specifically about the patient’s experiences with treatment, adherence to medication, and any setbacks, and stimulate therapy using motivational interviewing techniques. In comparison with usual care, the additional time for the MCAI is estimated at 10 min in total.

### Sequence generation blinding and masking

As mentioned previously, after training, nurses are able to employ the skills of shared decision-making and motivational interviewing in all patients. Accordingly, we have chosen a before-after study with two equal groups. First, patients are recruited for the control group. After sufficient inclusions, nurses are trained. Then, recruitment for the intervention group will start. This sequential design makes sequence generation and blinding redundant. Therefore, sequence generation, blinding and masking are not employed during the trial.

### Data collection, data quality and management

In compliance with the General Data Protection Regulation (GDPR) and the Dutch general data protection regulation (translated: Algemene Verordening Gegevensbescherming (AVG)), data is stored in Castor EDC [41], which is NEN7510- and ISO27001 certified. Patients are given the option to complete the surveys in hardcopy or digitally. All surveys are entered in Castor, either by the patients themselves or, in case of hard copy questionnaires, by the research team. All hardcopy surveys are coded and stored in a secured office in a locked cabinet. In order to ensure that all actions are conducted in the proper manner, Standard Operating Procedures (SOPs, fidelity to the recruitment procedures, contact information, survey preferences and comorbidities) are written in co-operation with the FLS nurses, physicians and researchers. The SOPs are provided to everyone involved in the study and are also located at the study sites. Each returned survey is assessed within two weeks for completeness and consistency.

### Outcomes

An overview of the primary and secondary outcomes, as well as covariates, can be found in Table 1.

**Table 1:** Outcomes measurements

Patient Outcomes	Instruments	Abbreviation	T0 (baseline)	T1 (4 months)	T2 (12 months)
**Primary outcomes**					
Medication persistence	Pharmacy refills (pharmacy)	–			x
**Secondary outcomes**					
Medication adherence	Pharmacy refills	–	x	x	x
Shared decision-making	Shared Decision-Making Questionnaire – 9 items	SDM-Q9	x	x	
	Decision Conflict Scale (DCS) - 16 items	DCS	x	x	
	Value of shared decision-making perceived by the patient	–	x	x	
Fractures and mortality	Hospital records Municipal Basic Administration (mortality)	–			x
Resource utilization and other costs	Self-developed cost-questionnaire	–	x	x	x
Health utility	EuroQoL 5-Dimension 5-Level	EQ-5D-5L	x	x	x
**Covariates**					
Health literacy	Health Literacy Survey-Europe, Q16 - 16 items	HLS-EU-Q16	x		
Comorbidties	Rheumatic Disease Comorbidity Index – 11 items	RDCI	x		
Socio-demographic characteristics	Self-developed Questionnaire	–	x	x	

### Primary outcomes

#### Medication persistence

Medication persistence is defined as the proportion of patients who are persistent at one year, based on actual delivery by the patients’ pharmacy. A refill gap of ≥30 days before the end of follow up is used to define non-persistence to oral medications; this is similar to previous studies [42–45]. Persistence is dichotomized as a) persistent at 12 months and b) not persistent at 12 months. We consider an absolute improvement of 20% in medication persistence to be clinically relevant.

### Secondary outcomes

#### Medication adherence

Medication adherence is defined as the percentage of days that patient was in possession of anti-osteoporosis medication, i.e. the mean possession rate (MPR) until the 12 months follow-up. Identification of the MPR will be based on actual delivery by the patients’ pharmacy. Adherence will be dichotomized as a) adherent after 12 months (MPR ≥ 80%) and b) not adherent after 12 months (MPR < 80%).

#### Decision quality

Decision quality entails whether a patient feels informed, certain regarding their decision, and experiences sufficient support at the moment the decision is made, regardless of the outcome of the treatment. Three questionnaires are administered to address this outcome domain. First, the 9-item Shared Decision Making Questionnaire (SDM-Q9) [46] is used to investigate the extent to which patients feel involved in the decision-making process. Second, the Decision Conflict Scale (DCS) [37] is used to investigate how comfortable patients feel regarding the decision made regarding their treatment. Finally, the extent to which patients appreciate shared decision-making for osteoporosis is obtained by two additional questions on a 7-point Likert scale: “How important is shared decision-making for you regarding treatment for your osteoporosis?” and “How do you feel regarding shared decision-making for your osteoporosis treatment?”. All three questionnaires are administered twice: at baseline and 3-4 months after baseline.

#### Fractures and mortality

The number of subsequent fractures and mortality are assessed 12 months after inclusion. The number of subsequent fractures are extracted from the medical hospital records and mortality from the municipal basic administration. In case the patient is no longer being treated in a participating hospital, the figure from the municipal basic administration is used.

#### Cost and health utilities

Data on health utilities and resource utilization are collected via questionnaires an adjusted questionnaire from the Maastricht study [47]: costs within the healthcare system (such as medication, diagnostic tests and other forms of resource utilization), costs for the patient and their relatives (such as informal care), costs for other sectors outside the healthcare sector (such as productivity losses) [48]. The costs of the intervention are comprised of (a) the extra time for the nurse for shared decision-making and motivational interviewing (b) costs of the DA. Health utility is assessed with the EuroQol 5 dimensions 5 levels (EQ-5D-5L) [39].

### Co-variates

#### Health literacy

In order to correct the effect of health literacy on medication persistence, health literacy, defined as the capacities of people to meet the complex demands of health in modern society [49] is assessed at baseline. The Health Literacy Survey-Europe, Q18 (HLS-EU-Q16) containing 16 questions which make a distension between four levels of health literacy: excellent, sufficient, problematic and insufficient is used.

#### Socio-demographic characteristics

Characteristics of the participants are used to describe the study population and, if needed, to correct for differences between the intervention and control group. Collected characteristics include: age, gender, education level, marital status, and occupational status (including other activities such as performing informal care and voluntary work).

#### Comorbidities

At baseline, the number of comorbidities including back and joint pain are collected by a member of the research team during the inclusion interview, using the Rheumatic Disease Comorbidity Index (RCDI) [50].

### Analysis

For all statistical analysis, the latest version of IMB SPSS is used. For null-hypothesis testing, *P*-values < 0.05 are regarded as clinically and statistical different.

### Primary outcome

#### Medication persistence

To compute between-group differences in the proportion of patients who are persistent at one year, we use multivariable logistic regression analysis adjusted for potential confounding variables, as group allocation is not randomized. Potential confounders include medication adherence, decision quality, fracture type, health utility and mortality.

The following outcomes need to be dichotomized: decision quality, educational level and number of comorbidities. To dichotomize these variables, the 50^th^ percentile is used. Since health literacy consists of three categories, this variable is dichotomized into a) insufficient and poor health literacy and b) sufficient health literacy. Both forward selection and backward elimination will be used to construct the model with the highest predictive value [51]. Since the outcome of a logistic regression can be influenced to a large extent by the choice of cut-off points, sensitivity analyses are conducted [52].

### Secondary outcomes

#### Medication adherence

A Chi^2^ test is used to identify differences between the intervention and control group in terms of adherence.

#### Decision quality

For decision quality, two outcomes: shared decision-making [46] and decision conflict [37] are assessed with independent T-tests. To assess differences in the extent to which patients feel comfortable and appreciate shared decision-making for osteoporosis, descriptive statistics are used.

#### Fractures and mortality

An independent a T-test is used to identify differences between the intervention and control group in terms of the number of fractures. A Chi^2^ test is used to identify differences between the intervention and control group in terms of the number of mortalities.

### Other outcomes

#### Health literacy

A Chi^2^ test is used to identify differences in persistence between the intervention and control group according to the level of health literacy.

#### Socio-demographic characteristics

To identify differences in persistence between the intervention and control groups according to socio-demographic characteristics, unpaired T-tests, Chi^2^ or ANOVA, are used as appropriate.

#### Comorbidities

Differences between the intervention and control groups in terms of the number of comorbidities are tested in two ways: unpaired T-tests for the total amount of co-morbidities and Chi^2^ for stratifying the number of co-morbidities.

### Economic evaluation

An economic evaluation in the form of a cost-utility analysis is conducted. Results are expressed as incremental lifetime cost per QALY gained of the MCAI compared to UC. A microsimulation Markov model developed in TreeAge (TreeAge Pro 2020, R2.1. TreeAge Software, Williamstown, MA) is used to simulate the lifetime natural history of Dutch patients with osteoporosis and is performed according to the Dutch guideline for economic evaluation and for costing, as well as the recent guideline of economic evaluations in osteoporosis from ESCEO-IOF [43–45]. Both univariate and probabilistic sensitivity analysis is conducted to handle uncertainty [53].

### Process evaluation

To assess whether the MCAI has been conducted as designed and to determine the feasibility of implementation of the intervention, a process evaluation is planned in which an emphasis on qualitative outcomes, such as the experiences of patient, nurses and medical staff. The process evaluation will be performed in line with the method described by Moore et al. [54]. This method contains three main components;

1. Implementation: evaluation of the intervention itself and how it has been delivered at the FLS (intervention fidelity).
2. Mechanisms of impact: evaluation of the effects of the intervention and factors responsible for the effectiveness.
3. Context: evaluation of the extent to which the setting has contributed to the effectiveness of the intervention.

## Discussion

This study aims to assess whether an MCAI is superior to usual care, in terms of medication persistence and cost-effectiveness for patients with osteoporosis who attend the FLS. This study has several strengths. First, the intervention is innovative, as it is grounded in principles of shared decision-making, but completed by motivational interviewing during the follow-up visit, to enhance medication persistence.

Further, cost-effectiveness and process evaluation are performed in addition. Cost-effectiveness is relevant, as FLS is high volume care and there is no room to implement care that is affordable when weighted against health benefits. The process evaluation is essential to interpret the trial results and provides useful information regarding the feasibility and implementablity of the MCAI at the FLS.

We expect that the results of this study are relevant to understanding (a) the real benefits of shared decision-making in the setting of an FLS and (b) whether an MCAI is able to improve persistence with an anti-osteoporosis drug. The study is conducted in the real-life setting of two established FLSs. By implementing the MCAI in the current daily practice of the FLS, it is expected MCAI can be adopted easily, and evaluated with a process evaluation, without substantially disrupting the daily routine of the outpatient clinics. The expected rapid inclusion eliminates the necessity of more participating centers. Furthermore, the involvement of both patients and professionals in the development of the MCAI ensures maximum participation of all parties involved. All the above contribute to ensure the best possible and affordable future care for osteoporosis.

Despite our best efforts, we foresee or expect the following limitations. First, medication persistence is used as a surrogate for medication adherence and as the primary outcome. Medication adherence is defined as the process by which patients take their medications as prescribed, and is composed of initiation, implementation and discontinuation [14]. It is difficult to translate the outcomes of a trial aiming to improve medication adherence with a dosing regimen to a real-life setting; once patients’ drug intake is monitored actively, it is expected that patients will alter their behavior. We expect this real-life data of prescription renewal will provide the most reliable data regarding medication persistence. Second, there is no consensus regarding data on current persistence with osteoporosis medication in the FLS in current literature, which makes it difficult to estimate baseline persistence. If the baseline persistence level is higher than the estimated baseline, (for example 60% higher), with the sample size of 248 patients, we can assess a significant difference of a smaller improvement in persistence (about 16-17%). We still regard this as a clinically relevant improvement, which will lead us towards the most effective interventions. Third, as a maximum of two nurses are working per FLS clinic, a consecutive random allocation is not feasible because of contamination problems. To avoid contamination of nurses’ knowledge about the DA and the adherence support program after training, the first patients are assigned to the control group and sequentially, when the targeted sample size for the control group is reached, new participants are assigned to the intervention group. Fourth, albeit we are aware that our sample size has not been calculated on a post-hoc analysis, we acknowledge the importance of this; identification of relevant subgroups or preferences can be relevant in proposing patient-specific treatment [21]*.*

In conclusion, we expect that the ICON study will show that the MCAI is a (cost-)effective and easily implemented intervention that could result in an improvement in the persistence with anti-osteoporosis medication, leading to fewer subsequent fractures, lower mortality and a higher quality of life. Moreover, while nurses play a key role in the organization and daily performance of an FLS and are therefore highly suitable for testing the MCAI [36], we are convinced that the MCAI could be used by other healthcare professionals, such as rheumatologists and general physicians, making it a widely applicable intervention.

### Trial status

On September 1, 2018, inclusion of the patients was initiated in the first center and May 2019 in the second center. The inclusion of the last 25 patients was delayed due to the consequences of COVID-19 pandemic on available personnel, somewhat lower fracture rates and reduced attendance of patients. We expect to finalize the inclusion of the control group at the end of June 2021. After all patients from the control group have had their 3-month follow-up, nurses will be trained in the usage of the DA and motivational interviewing, and the inclusion of the intervention group will then start, and is expected to take place between September and December 2021. After the 12 month-follow-up of the final patients in December 2022, data analysis is expected in early semester 2023.

## Data Availability

Five years after completion of the follow-up, the datasets generated and/or analyzed during the current study will be available from the corresponding author on reasonable request.

## Abbreviations

AVG: Algemene Verordening Gegevensbescherming (Dutch, translated; General Data Protection Regulation)
CEA: Cost-Effectiveness Analysis
CRF: Clinical Report Form
CUA: Cost Utility Analysis
DA: Decision Aid
DCS: Decision Conflict Scale
DXA: Dual-energy X-ray absorptiometry
EQ-5D-5L: EuroQol, 5 dimensions, 5 levels
FLS: Fracture Laison Service
GDPR: General Data Protection Regulation
HLS-EU-Q16: Health Literacy Survey Europe, 16 questions
QALY: Quality Adjusted Life Years
MCAI: Multi Component Adherence Intervention
METC: Medisch Ethisch Toetsings Commissie (Dutch, translated: Medical Ethical Committee)
SDM-Q9: Shared Decision Making-Questionnaire 9 Questions
SOP: Standard Operating Procedure
RCDI: Rheumatic Disease Comorbidity Index
